# Design and Analysis of Non-Binary LDPC-CPM System for Hybrid Check Matrix Construction Algorithm of WSN

**DOI:** 10.3390/s18082418

**Published:** 2018-07-25

**Authors:** Jiahui Meng, Danfeng Zhao, Liang Zhang

**Affiliations:** College of Information & Communication Engineering, Harbin Engineering University, Harbin 150001, China; zhaodanfeng@hrbeu.edu.cn (D.Z.); zhliang3610@hrbeu.edu.cn (L.Z.)

**Keywords:** wireless sensor network (WSN), non-binary low-density parity check (LDPC), iterative coding algorithm, hybrid check matrix construction (HC) algorithm, LDPC-CPM system

## Abstract

In order to enhance the reliability and anti-interference performance of wireless sensor network (WSN) data transmission, this paper designs the low power scheme of the WSN from the angle of error correction coding and proposes the hybrid check matrix construction (HC) algorithm based on iterative coding algorithms with linear coding complexity. The algorithm first improves the traditional iterative coding algorithm, making it suitable for non-binary low-density parity check (LDPC) codes. Then, the algorithm applies the backward iteration method to change the coding scheme and uses the check matrix construction method so that the progressive edge growth (PEG) algorithm has a lower triangular structure, which is used as a base matrix. An improved quasi-cyclic LDPC (QC-LDPC) algorithm, with a lower triangular structure, is used to generate a cyclic shift matrix and a finite domain coefficient matrix. Simultaneously, the short loop is eliminated and the optimal check matrix is selected for use in the channel coding process. The non-binary LDPC-CPM system is modeled and simulated. The simulation results show that the non-binary LDPC code constructed by the HC algorithm not only has linear coding and storage complexity but also has strong error correction capability. The design of non-binary LDPC-CPM system parameters can enhance the reliability, anti-jamming capability and reduce the complexity and reduce the complexity of the WSN.

## 1. Introduction

The wireless sensor network (WSN) is a network information system integrating distributed information acquisition, information transmission and information processing technologies [1,2,3,4]. It adopts a self-organizing method to configure a large number of sensor nodes and collects and processes the target information in the network coverage area through the collaborative work of the nodes. Different from traditional wireless network to improve service quality and efficient bandwidth utilization, this paper adds coding and decoding technology to WSNs, which can greatly improve the anti-noise performance and improve communication quality of WSNs. Low power consumption is especially important for WSN nodes during the design process. Since the energy consumption of the WSN nodes is mainly concentrated in the computing module and the communication module, in addition to adopting dynamic energy management measures for them, the innovation of this paper is to add a non-binary LDPC-CPM coding and decoding module in the traditional WSNs node communication module. In order to improve the data error correction performance of data transmission between sensor nodes and between nodes and aggregation nodes, the WSNs data transmission reliability and WSNs anti-interference performance are enhanced. Highly reliable data transmission can reduce the communication traffic sent and received by the communication module, thereby reducing the energy consumption of the communication module. In this paper, the design of the non-binary LDPC-CPM scheme in WSNs mainly includes the selection of linear block code, coding algorithm, check matrix construction algorithm, decoding algorithm, modulation and demodulation mode and so on.

In error correction coding, non-binary LDPC codes are the best ones currently used for error correction capability, low coding complexity and high decoding efficiency and are very suitable for use in WSN [5,6,7,8]. The research shows that the non-binary LDPC code using the belief propagation (BP) algorithm has excellent performance approaching the Shannon limit. LDPC code description is simple, with greatly flexibility and lower error leveling characteristics. Parallel operation, high throughput and high-speed decoding potential have been considered as a class of channel coding schemes comparable to Turbo codes [9,10,11,12]. The low complexity means that the power consumption is lower for practical working systems and thus it is very suitable for hardware implementation on WSN nodes [13,14,15]. The non-binary LDPC code can effectively reduce the transmit power received by the sensor node, thereby reducing the energy consumption of a single node. Therefore, the error correction coding scheme introduced in the WSN uses a non-binary LDPC code for a series of coding and decoding processes.

The WSNs node is a low-power, low-price micro-embedded device. Its energy supply and wireless communication bandwidth are very limited and the calculation and storage requirements are very high, so the requirements for the type of operation are also high. Therefore, the coding algorithm and check matrix construction algorithm in the non-binary LDPC-CPM scheme proposed in this paper are improved to reduce the computational complexity. In the non-binary LDPC encoding algorithm, the encoding algorithm based on Gauss elimination [16] and efficient encoding algorithm based on the approximate triangular structure (also called greedy algorithm) are suitable for the general check matrix but the check matrix needs to be pretreated in encoding process. The two algorithms all contain the inverse of the matrix. The process of calculating the inverse is not only a large amount of computation, hard to realize the hardware but also full rank of the matrix. In the actual application process, the above conditions cannot be met. In 2004, Wang Peng proposed an iterative encoding algorithm based on binary LDPC codes [17]. This algorithm has linear coding complexity and does not change the sparseness of the matrix. It also overcomes the disadvantages of inflexible code length and code rate selection. This paper improves the iterative encoding algorithm and transforms it into a coding algorithm for non-binary LDPC codes. It is called a non-binary iterative coding algorithm.

According to the structural characteristics of non-zero element distribution in the LDPC code check matrix, the construction method for LDPC code is divided into a random construction algorithm and structured construction algorithm. In 2005, Hu et al. proposed the progressive edge growth (PEG) random construction algorithm [18]. The check matrix constructed by this algorithm has a large girth, a small Hamming distance and large randomness. The decoding performance is good but the encoding complexity is high. This paper proposes an improved PEG (irPEG) algorithm to reduce the high encoding complexity of the PEG algorithm. The structured code construction algorithm, QC-LDPC [19], creates a corresponding check matrix based on a certain matrix structure. The QC-LDPC construction algorithm is relatively complex and decoding performance is worse than the random construction algorithm but the encoding complexity is significantly reduced.

To reduce the complexity of constructing the check matrix and accelerate the convergence speed, this paper introduces an improved QC-LDPC (ir-qc) algorithm, with a lower triangle structure in the check matrix. Using the ir-qc algorithm, a check matrix without short loops was constructed. Then, with a focus on encoding complexity and error correction performance and based on the improved non-binary superposition coding algorithm with linear encoding complexity, a hybrid construction (HC) algorithm is proposed. Combining random construction and structured construction algorithms, the irPEG algorithm constructs a base matrix and then uses the ir-qc algorithm to generate a cyclic shift matrix and a finite field coefficient matrix, eliminating the influence of the fourth and sixth rings. The number of check matrices constructed is set, from which the optimal check matrix is chosen. This algorithm not only has the randomness of a random construction algorithm but also maintains the low encoding complexity of the structured construction algorithm and reduces the loss in the error caused by the structured construction. HC algorithm is a comparative compromise algorithm.

It can be seen that the non-binary LDPC code has a good application prospect in a WSN requiring high reliability, low delay and high rate. Moreover, the optimization of the non-binary LDPC-CPM scheme can effectively reduce the energy consumption and computational complexity of the WSN. Therefore, this paper mainly optimizes the non-binary LDPC-CPM scheme in WSNs from four aspects: improving system reliability, reducing computational complexity, improving error correction performance and reducing energy loss.

## 2. Design of WSNs with Non-Binary LDPC Coding

### 2.1. WSNs Basic Structure with Non-Binary LDPC Coding

**Figure 1:**
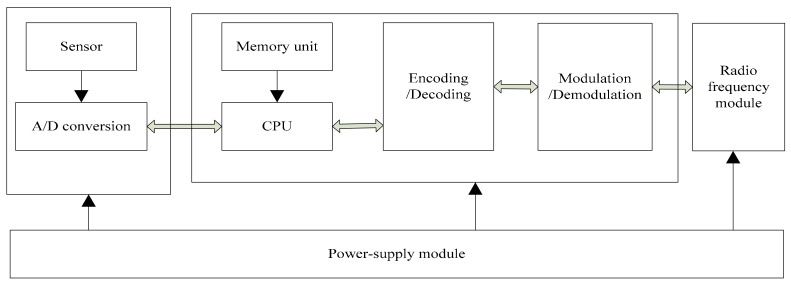
Node structure of wireless sensor.

Channel coding systems are rarely used in WSNs but Cyclical Redundancy Check (CRC) is used. However, the CRC is characterized by intelligent identification errors and failure to complete error correction. Once an error occurs during the transmission, a retransmission is required. Therefore, when the amount of data transmitted each time is slightly larger, the distance is slightly longer and the real-time requirement is higher, the retransmission rate will be very high, so this method of error retransmission will be a big disadvantage. When the probability of information error in the process transmitting data is very high, the link reliability needs to be improved. Therefore, the coding and decoding module of the non-binary LDPC code is added to the communication of the traditional sensor node to improve the data error correction performance of data transmission between sensor nodes, nodes and aggregation nodes, as shown in Figure 1.

### 2.2. The Basic Principles of WSNs Coding with Non-Binary LDPC Codes

In the sensor network, the physical layer is the key to determine the volume, cost and energy consumption of the nodes of the sensors network [20,21,22]. Because of the low capability of WSNs node processors and relatively small storage space, the design should have the following characteristics:

1. Designing non-binary LDPC codes with relatively short code length.

WSN energy supply and wireless communication bandwidth is very limited, the size of the node is small, the processing speed of the microcontroller in the node is relatively low, the ability to process data is low and most of the nodes are powered by the battery. Although academia is studying renewable energy and automatic charging mechanisms, too small a node is still a factor limiting its application. Therefore, this paper designs a non-binary LDPC code with a relatively short code length to suit the processor capabilities of the WSN node. Considering the comprehensive advantages of the short-code and low-complexity of the construction of the hybrid parity check matrix construction algorithm, this paper adopts the hybrid method to construct the check matrix.

2. Because of the check matrix H of non-binary LDPC code is very large, we use reasonable storage technology to store check matrix H and generate matrix G.

The storage space of WSN nodes is relatively small but the check matrix dimension is large, so it is impossible to store the entire check matrix. The solution to this problem is to store only the position of non-zero elements in the base matrix constructed by the irPEG algorithm and its corresponding cyclic shift matrix in each row or column of the check matrix.

3. Selection of modulation methods suitable for WSNs.

In order to improve the frequency band utilization of WSNs and reduce redundant information, continuous phase modulation (CPM) is used to form a non-binary LDPC-CPM system. CPM is an advanced phase modulation technology with continuous phase characteristics and excellent spectral characteristics. Compared with the phase-shift keying (PSK) modulation method, CPM has a higher frequency band utilization ratio. Moreover, CPM modulation system is a combination of channel coding and modulation. By controlling the state transition at the next moment by generating the phase state lattice sequence, the modulation of the information symbol has a coding effect directly, so that no more redundant symbols are needed.

4. On the basis of satisfying certain performance, we use the algorithm of encoding and decoding with loss computation and at the same time, the number of bits of data quantization is less.

Because WSN node energy supply and communication bandwidth are limited and the processing speed of the microcontroller in the node is relatively low. Therefore, a non-binary LDPC code encoding and decoding scheme with good error correction performance and low algorithm complexity should be selected. According to the previous analysis, the choice of iterative encoding algorithm and Log-FFT-BP decoding algorithm [23,24] is relatively suitable for the requirements of WSN nodes.

5. The number of iterations is as few as possible.

The number of decoding iterations is an important factor influencing the decoding performance of a non-binary LDPC code. When the number of iterations is too small, error correction performance will be reduced but too high number of iterations will increase the amount of decoding operations. Therefore, the non-binary LDPC codes applied to the WSN should be chosen in combination with the actual situation to select the appropriate number of decoding iterations.

## 3. Non-Binary Iterative Encoding Algorithm

**Figure 2:**
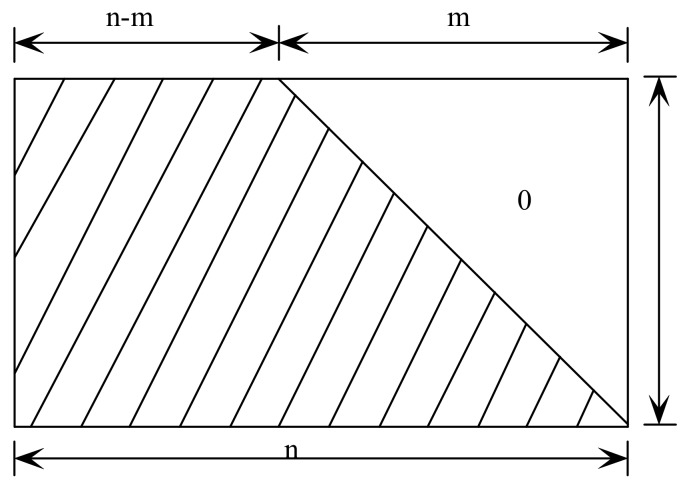
Check matrix with lower triangle structure.

The Gaussian elimination coding algorithm is the most basic coding algorithm in LDPC codes. Like the binary LDPC code, the non-binary LDPC code is also applicable to the Gaussian elimination coding algorithm. The advantage of Gaussian elimination coding algorithm is that the coding principle is simple but the computational complexity is large and the computational complexity is high. Therefore, the non-binary iterative coding algorithm is studied in this paper [25,26,27]. If the check matrix of the non-binary LDPC code has a lower triangular structure as shown in Figure 2, the elements on the diagonal lines are all non-zero elements on the Galois Field and the remaining non-zero elements are all on the left side of the diagonal, iterative encoding algorithm can be used directly for encoding.

Set the code word vector as $c\in F^{n}$, divide it into two parts, namely information vector $s\in F^{n-m}$ and check bit vector $p\in F^{m}$, namely c=(s,p), then the encoding algorithm is specifically shown in Equation (1).

(1) $$p_{l}=\left(\sum_{i=0}^{k-1}h_{i,j}\cdot s_{j}+\sum_{i=0}^{l-1}h_{i,j+k}\cdot p_{j}\right)/h_{l,k+l}$$

Among them, l∈[0,n−k−1], $h_{i,j}$ represents the element on the *i*-th row and *j*-th column in the check matrix H and k=n−m. According to Equation (1), the process of the encoding algorithm is to use the constraint relationship between the rows in the check matrix H to calculate the symbol value of each check bit in turn using the backward iterative method.

The binary iterative encoding algorithm is improved and AND and XOR operations used in binary are improved to be multiplication and addition operations in Galois Field. In order to simplify the calculation amount, set all the elements on the diagonal to be 1 and in this case, Equation (1) is improved to Equation (2).

(2) $$p_{l}=\sum_{i=0}^{k-1}h_{i,j}\cdot s_{j}+\sum_{i=0}^{l-1}h_{i,j+k}\cdot p_{j}$$

When w can be regarded as a small constant with respect to n, the non-binary iterative encoding algorithm has a linear complexity, where n is the code length and w is the average column weight of the generated matrix.

## 4. Hybrid Check Matrix Construction (HC) Algorithm

### 4.1. IrPEG Construction Algorithm

The check matrix was constructed prior to decoding. Given the disadvantage of the higher encoding complexity of the existing PEG algorithm, this paper proposes an improved non-regular PEG algorithm with a lower triangle structure based on an improved non-binary superposition coding algorithm, namely the irPEG construction algorithm. The algorithm is improved from two aspects: encoding scheme and constructing check matrix, reducing the complexity of the algorithm and improving error correction performance.

Since the PEG algorithm is a random construction algorithm, each time the non-zero elements on the Galois field are filled, the position of the fill is random [28,29]. To reduce the encoding complexity of the check matrix construction algorithm, a base matrix with a lower triangular structure was constructed using specified bit node degree distribution, as shown in Figure 2. Compared with the PEG algorithm, the check matrix constructed by the irPEG algorithm has a certain structural constraint and then the values of all check node positions were determined by adopting the backward superposition method. The specific steps of the irPEG algorithm are as follows. (1) Determine the parameters in the base matrix: the number of rows and columns and the sequence of variable node degrees and initialize the base matrix information, including the set of check nodes connected to the variable node and its complement. (2) Construct the lower triangular part on the right side of the base matrix.

First, the last variable node was constructed using the backward iteration method and the check nodes were connected forward according to the degree sequence of the variable node. The position of the first non-zero element in each column had to be connected to the check node on the diagonal and the remaining non-zero elements were added below the diagonal, which can be used with the linear encoding complexity of the improved non-binary superposition coding algorithm for encoding. The benefit of the irPEG algorithm is that it has a lower encoding complexity than the PEG algorithm. Then, all the check node sets connected with the variable node were searched and the check node set with the smallest degree was filtered out. If the set contained multiple elements, the check nodes that could form four rings and six rings were deleted. Finally, one of the remaining check nodes was randomly connected and if there was only one element, the node was directly connected. If the set contained multiple elements, the check nodes that could form four rings and six rings were deleted Finally, one of the remaining check nodes was randomly connected and if there was only one element, the node was directly connected.

Construct the first n−m columns of the base matrix. The selection is constructed forward from the n−m variable node and the constructed method is similar to the lower triangle. Select the check node with the smallest degree according to the degree sequence of the variable nodes to ensure that the row weight of each row is not considerably different from the average row weight. After the check nodes from the fourth ring are deleted, the connection is randomly selected from the remaining check nodes.

(3) $$H=\left[\begin{array}{cccccccccccccccc} 0 & 3 & 0 & 0 & 7 & 0 & 4 & 13 & 1 & 0 & 0 & 0 & 0 & 0 & 0 & 0\\ 0 & 0 & 3 & 3 & 11 & 1 & 0 & 0 & 14 & 1 & 0 & 0 & 0 & 0 & 0 & 0\\ 11 & 0 & 0 & 0 & 0 & 0 & 0 & 0 & 6 & 12 & 1 & 0 & 0 & 0 & 0 & 0\\ 14 & 3 & 0 & 0 & 0 & 0 & 3 & 0 & 0 & 7 & 7 & 1 & 0 & 0 & 0 & 0\\ 0 & 5 & 8 & 8 & 0 & 0 & 0 & 0 & 14 & 0 & 0 & 1 & 1 & 0 & 0 & 0\\ 0 & 0 & 0 & 0 & 0 & 7 & 0 & 3 & 0 & 0 & 0 & 0 & 12 & 1 & 0 & 0\\ 13 & 0 & 7 & 7 & 1 & 0 & 10 & 0 & 0 & 0 & 0 & 0 & 0 & 8 & 1 & 0\\ 9 & 0 & 0 & 0 & 8 & 7 & 0 & 0 & 0 & 0 & 12 & 0 & 0 & 0 & 4 & 1 \end{array}\right]$$

Since the irPEG algorithm belongs to a random check matrix construction algorithm and the constructed check matrix has no certain structure, the hexadecimal $H_{8\times 16}$ of Equation (3) is used as an example to illustrate the structure of the proposed check matrix constructed and improved irPEG algorithm. The degree distribution of the bit nodes is [30,31]:

(4) $$\lambda \left(x\right)=0.38354x+0.04237x^{2}+0.57409x^{3}$$

Since the constructed matrix has a lower triangular structure and is constructed to satisfy the degree distribution of Equation (4), the column weight of the last column of the matrix is set to 1 and the elements of the check section on the diagonal line are all 1. The elements of the lower triangle are all 0. The improved non-binary superposition coding algorithm can be directly performed by using Equation (2).

### 4.2. Hybrid Construction (HC) Algorithm

The PEG algorithm belongs to the construction algorithm of the randomly constructed non-binary LDPC code check matrix. Although its error correction performance is superior to the structure construction algorithm because of the randomness of the constructed matrix, the hardware implementation complexity is higher than the structure construction algorithm. Although the proposed irPEG algorithm combined with the superposition coding algorithm can reduce the encoding complexity of non-binary LDPC codes, it is more suitable for hardware implementation of short codes in non-binary LDPC codes, as the hardware implementation complexity for long codes is still high. At this time, through the loss of certain error correction performance in non-binary LDPC codes, the random construction algorithm and structure construction algorithm of non-binary LDPC codes check matrix are combined and the complexity of the hardware implementation is reduced based on the superposition coding algorithm and a hybrid construction algorithm is proposed. Based on the ir-qc construction algorithm, the hybrid construction algorithm has the structural characteristics of the lower triangles. Simultaneously, the irPEG algorithm is used to construct the binary base matrix $W_{J\times L}$, which improves the randomness of non-binary LDPC codes and reduces the loss of error performance due to structured construction. The specific steps of the HC algorithm are as follows:

- The irPEG algorithm constructs the binary base matrix $W_{J\times L}$ . For a given degree distribution of a non-binary LDPC code, a binary matrix with a lower triangular structure is constructed according to the irPEG algorithm and the size is J×L.
- Determine the finite field element coefficient matrix $Gc_{J\times L}$ and randomly select the value of $gc_{j,l}$ between (0,q−1) according to the position of the non-zero elements in the base matrix, where $q=2^{b}$.
- From the binary base matrix $W_{J\times L}$, determine the cyclic shift coefficient matrix $S_{J\times L}$.

Although the binary base matrix $W_{J\times L}$ has a lower triangular structure, after cyclic shift, the check matrix does not necessarily have a lower triangular structure. To ensure the structure always has a lower triangle, the coefficients on the diagonal line of the cyclic shift coefficient matrix were set to 0 and thus, the check matrix constructed has a lower triangular structure. The principle of randomly selecting shift coefficients $s_{j,l}$ in the shift coefficient matrix $S_{J\times L}$ was to avoid the short loops in the Tanner graph as much as possible. The necessary and sufficient conditions for avoiding the short loop of length $2^{i}$ through the binary base matrix $W_{J\times L}$ combination are shown in Equation (5). A continuous heuristic was used to determine the shift coefficients $s_{j,l}$ in the shift coefficient matrix $S_{J\times L}$ at the non-zero elements of the binary base matrix $W_{J\times L}$.

(5) $$\sum_{k=1}^{m}\left(s_{j_{k},l_{k}}-s_{j_{k+1},l_{k}}\right)\neq 0\mathrm{mod}p$$

**Figure 3:**
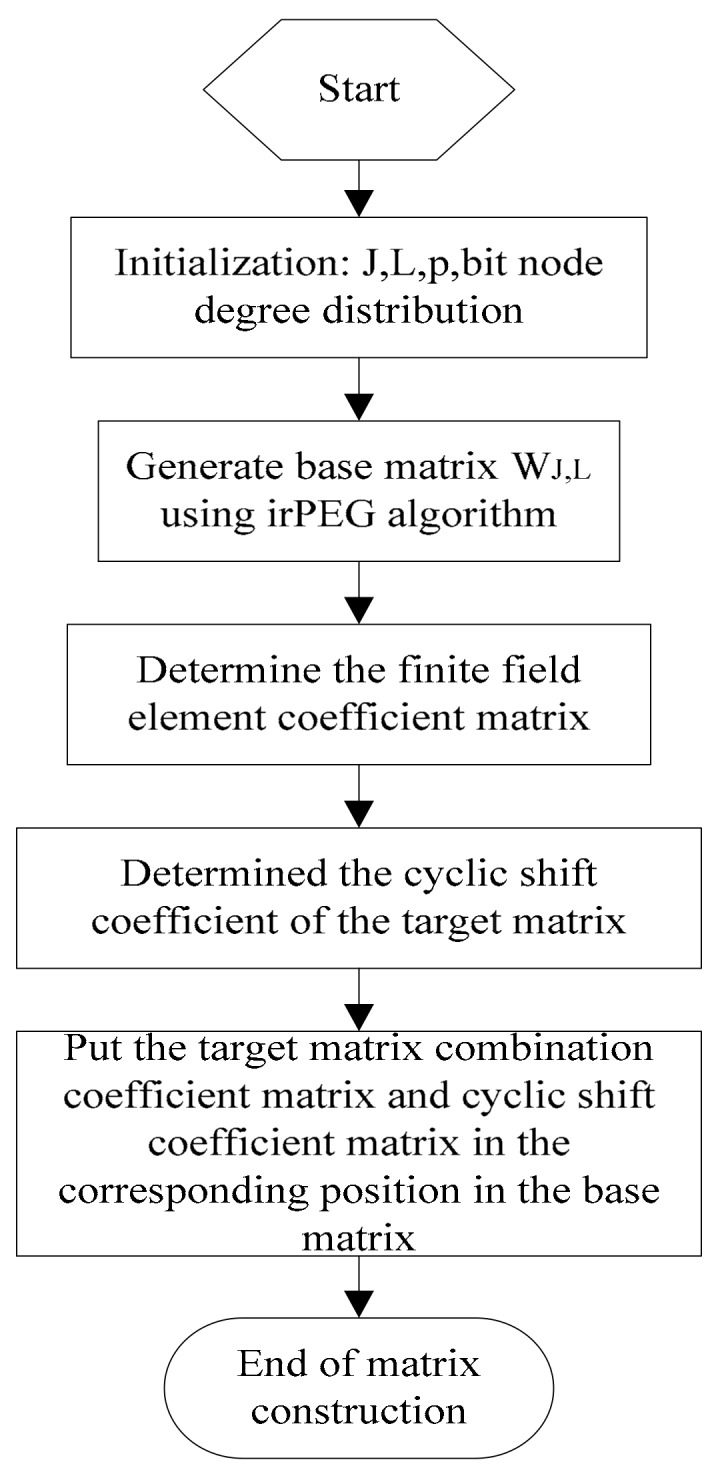
Flow chart of the hybrid check (HC) matrix check algorithm.

1. First, determine the target square P of the binary p×p dimension, as shown in Equation (6). If the value of the element $gc_{j,l}$ in the *j*th row and the l-column of the coefficient matrix $Gc_{J\times L}$ is not “0,” that is, $gc_{j\times l}\neq 0$, 1≤j≤J and 1≤l≤L. Randomly select a value of the shift coefficient $s_{j,l}$ from the set {0,1,⋯,p−1}. For a given degree distribution, according to Equation (5), determine whether the constraint condition is satisfied. If so, the value of the shift coefficient $s_{j,l}$ can be determined and added to the cyclic shift coefficient matrix $S_{J\times L}$, if the constraint condition is not satisfied, then return to step 1.
  (6) $$P_{p\times p}=\left[\begin{array}{ccccc} 1 & 0 & 0 & \cdots & 0\\ 0 & 1 & 0 & \cdots & 0\\ \vdots & \vdots & \vdots & \ddots & \vdots \\ 0 & 0 & 0 & \cdots & 0\\ 0 & 0 & 0 & \cdots & 1 \end{array}\right]$$
2. If the value of the elements of the *j*th row and the l-column of the coefficient matrix $Gc_{J\times L}$ is “0,” that is, $gc_{j\times l}=0$, 1≤j≤J and 1≤l≤L, then $s_{j,l}=\infty$. To summarize, the form of the cyclic shift coefficient matrix $S_{J\times L}$ is as shown in Equation (7).
  (7) $$S_{J\times L}=\left[\begin{array}{ccccccc} s_{1,1} & \cdots & s_{1,L-J} & 0 & 0 & \cdots & 0\\ s_{2,1} & \cdots & s_{2,L-J} & s_{2,L-J+1} & 0 & \cdots & 0\\ \vdots & \ddots & \vdots & \vdots & \vdots & \ddots & \vdots \\ s_{J,1} & \cdots & s_{J,L-J} & s_{J,L-J+1} & s_{J,L-J+2} & \cdots & 0 \end{array}\right]$$
3. The check matrix H is constructed using the binary base matrix $W_{J\times L}$, the cyclic shift coefficient matrix $S_{J\times L}$ and the coefficient matrix $Gc_{J\times L}$. If $w_{j,l}=0$, the sub-matrix $H_{j,l}=0_{p\times p}$, that is, $H_{\left(\left(j-1\right)p+1:jp\right),\left(\left(l-1\right)p+1:lp\right)}=0_{p\times p}$. If $w_{j,l}=1$, then $H_{j,l}=gc_{j,l}P_{p\times p}\left(s_{j,l}\right)$, that is, $H_{\left(\left(j-1\right)p+1:jp\right),\left(\left(l-1\right)p+1:lp\right)}=gc_{j,l}P_{p\times p}\left(s_{j,l}\right)$. The check matrix H is in the form of Equation (8).
  (8) $$H=\left[\begin{array}{ccc} H_{1,1} & \cdots & H_{1,L-J}\\ H_{2,1} & \cdots & H_{2,L-J}\\ \vdots & \ddots & \vdots \\ H_{J,1} & \cdots & H_{J,L-J} \end{array}|\begin{array}{cccc} P & 0 & \cdots & 0\\ H_{2,L-J+1} & P & \cdots & 0\\ \vdots & \vdots & \ddots & \vdots \\ H_{J,L-J+1} & H_{J,L-J+2} & \cdots & P \end{array}\right]$$
  where 0 represents the zero matrix of the p×p dimension, P represents the unit matrix of the p×p dimension, the code length is n=p×L and the coding rate is r=(1−J/L). The flow chart of the HC algorithm is shown in Figure 3.

## 5. Simulation Results and Analysis

**Figure 4:**
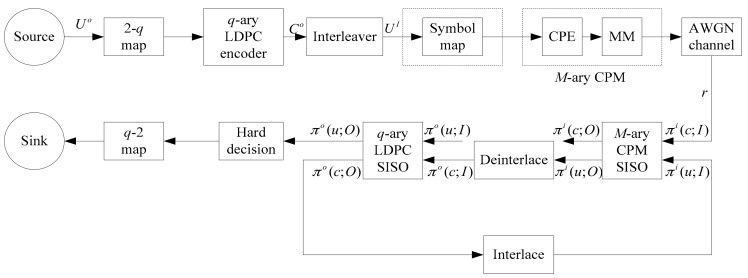
Non-binary LDPC-CPM system model.

Based on the memory and recursion characteristics of continuous phase encoder (CPE), combined with the external non-binary LDPC codes and random interleaver, the serial cascaded non-binary LDPC-CPM model is constructed [32], as shown in Figure 4. At the transmitter side, the dimension of the check matrix of the non-binary LDPC code is m×n and the length of the non-binary LDPC code is n, the bit of information is k=n−m and the code rate is r=k/n=1−m/n. The information sequence $U^{o}$ gets the code word sequence $C^{o}$ after mapping and non-binary LDPC coding and then through interleaving and symbol mapping into the M-ary CPM, where CPM is decomposed into CPE and memoryless modulation (MM) [33], CPE has continuous phase encoding of the input information as input of memory modulation MM and MM selects a suitable waveform to send to the channel. The dimension of the non-binary LDPC encoder and the higher-order CPM modulator is equal, that is, q=M.

The demodulation and decoding process is accomplished by the two subsystems M-ary Continuous Phase Modulation Soft-Input Soft-Output (CPM-SISO) iteration (called “external iteration”). The M-ary CPM-SISO subsystem uses the Log-MAP algorithm, the q-ary LDPC-SISO subsystem uses the Log-FFT-BP iterative decoding algorithm and the iteration is called the internal iteration. $\pi^{i}(c;I)$ and $\pi^{i}(u;I)$ are input probability sequences of the M-ary CPM-SISO subsystem, respectively and $\pi^{i}(c;O)$ and $\pi^{i}(u;O)$ are output probability sequences of code words and information words of M-ary CPM-SISO subsystem; $\pi^{o}(c;I)$ and $\pi^{o}(u;I)$ are input probability sequences of code words and information words of the q-ary LDPC-SISO subsystems and $\pi^{o}(c;O)$ and $\pi^{o}(u;O)$ are output probability sequences of q-ary LDPC-SISO subsystem code words and information words, respectively. After the information words probability sequence $\pi^{i}(u;O)$ output by the M-ary CPM-SISO subsystem is deinterleaved, it is used as the input probability sequence $\pi^{o}(c;I)$ of the q-ary LDPC-SISO subsystems code words and the output code words probability sequence of the q-ary LDPC-SISO subsystem. After being interleaved, $\pi^{o}(c;O)$ is input into M-ary CPM-SISO subsystem as the input word probability sequence $\pi^{i}(u;I)$ and the process is iterated repeatedly several times. The final iteration result is output by the q-ary LDPC-SISO subsystem as a hard decision.

In order to test the performance of the designed non-binary iterative detection receiver, a non-binary LDPC-CPM system with different parameters is simulated. The check matrix construction algorithm, code length, code rate, number of outer iterations and number of inner iterations of the non-binary LDPC code are designed. In the simulation, the Log-FFT-BP algorithm is used for decoding. The distribution of variable node degrees for irregular non-binary LDPC code is $\lambda \left(x\right)=0.38354x+0.04237x^{2}+0.57409x^{3}$; the CPM signal is for complex base band mapping. Each symbol is sampled at 8 to 10 points and the modulation index h is 1/2. The correlation length L is 2, the pulse function g(t) is Resistance-Capacitance (RC) type, that is, the specific signal form of CPM is OM2RC; the channel model adopts Additive White Gaussian Noise (AWGN) model. Error correction performance is detected using an Error-Rate Detector (ERD). ERD is a test instrument that generates a pseudo-random analog signal and compares it with the transmitted-received signal to calculate the error correction performance of the transmission medium. In this paper, a pseudo-random function is used in the simulation to generate a series of pseudo-random numbers, which are input into the encoder to obtain the coded code word and the corresponding probability information is obtained under the analog channel and input to the decoder for decoding. As a result, the decoded result is compared with the originally input code word to obtain the bit error rate of this paper.

### 5.1. A Check Matrix Construction Algorithm for Non-Binary LDPC-CPM System in WSNs

Firstly, the proposed two check matrix construction algorithms are simulated to find a check matrix construction algorithm suitable for WSNs. The simulation parameters of the irPEG algorithm are set as follows: The degree distribution is subject to the non-binary LDPC code of Equation (4) and the matrix is generated by the PEG algorithm and the irPEG algorithm, respectively. The simulation is carried out at hexadecimal 1/2 code rate (Code1) and hexadecimal 3/4 code rate (Code2) and the fixed information bit length. In Code1, the information bit length is 512 bits, that is, the symbol length is 128; In Code2, the information bit length is 176 bits, that is, the symbol length is 44.

**Figure 5:**
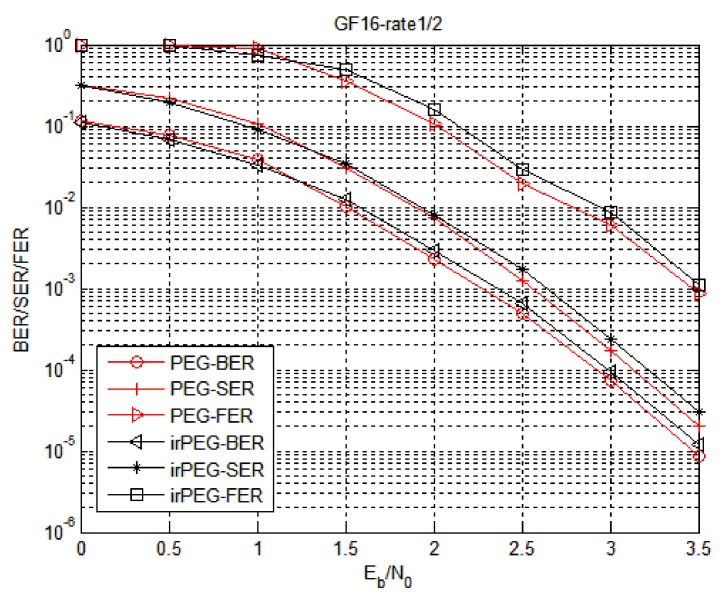
The error correction performance of improved progressive edge growth (irPEG) algorithm in Code1.

**Figure 6:**
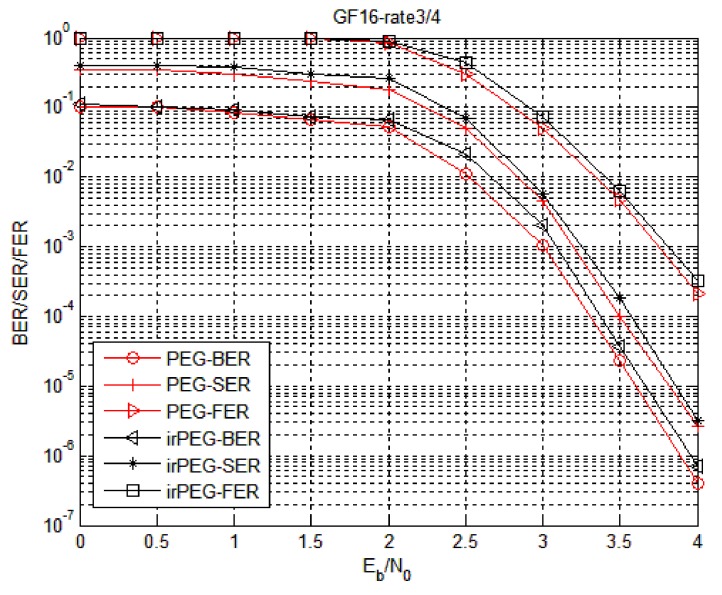
The error correction performance of irPEG algorithm in Code2.

Figure 5 and Figure 6 show the error correction performance of the irPEG and PEG algorithms at different code rates for Code1 and Code2, respectively. Compared with the non-binary LDPC code constructed by the irPEG and PEG algorithms, the error correction performance was not much different. The non-binary LDPC code with lower triangle structure constructed by the irPEG algorithm has a stronger error correction ability while considerably reducing hardware implementation complexity.

When the $E_{b}/N_{0}$ is 3 dB, the time required for decoding was calculated under the conditions of the code rate 1/2 Code1 and the code rate 3/4 Code2 respectively and the consistency of the simulation environment was maintained, which meant excluding the influence of unrelated factors during the simulation. The obtained data are shown in Table 1 and Table 2. From Table 1, under the same simulation environment, the time required by the irPEG algorithm was obviously less than the time required by the PEG algorithm. When the number of erroneous bits was small, the time saving was less than 50% but as the number of erroneous bits increased, the time saving was stable at 50%. Therefore, from the simulation results of Code1, the time spent by the irPEG algorithm was only 50% for the PEG algorithm. The simulation test result of Code2 when $E_{b}/N_{0}$ is 3 dB is shown in Table 2. The test results also showed that the time required for decoding was reduced by half.

**Figure 7:**
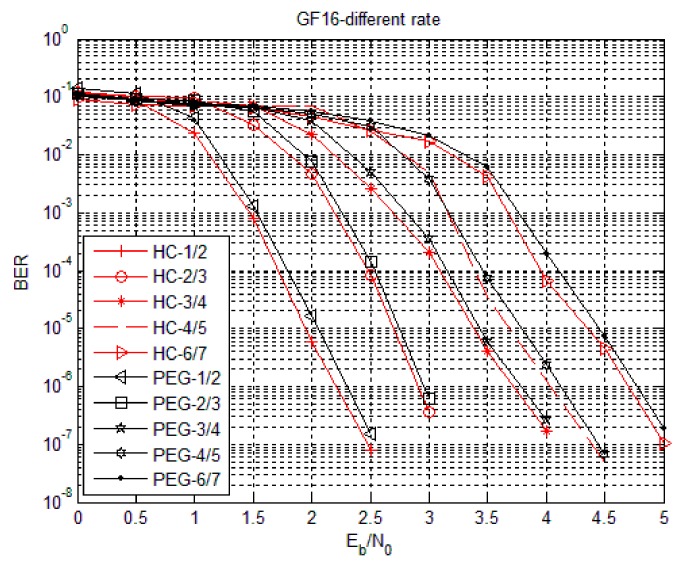
Bit Error Rate (BER) performance of HC algorithm under Code3.

**Figure 8:**
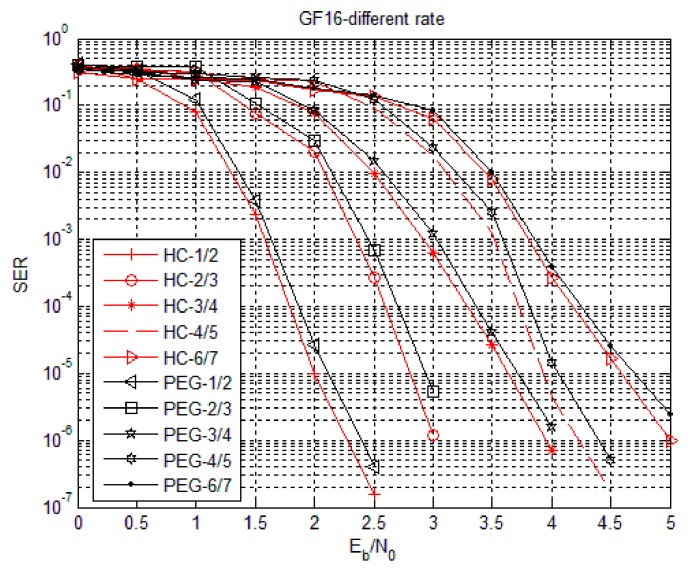
Symbol Error Rate (SER) performance of HC algorithm with Code3.

**Figure 9:**
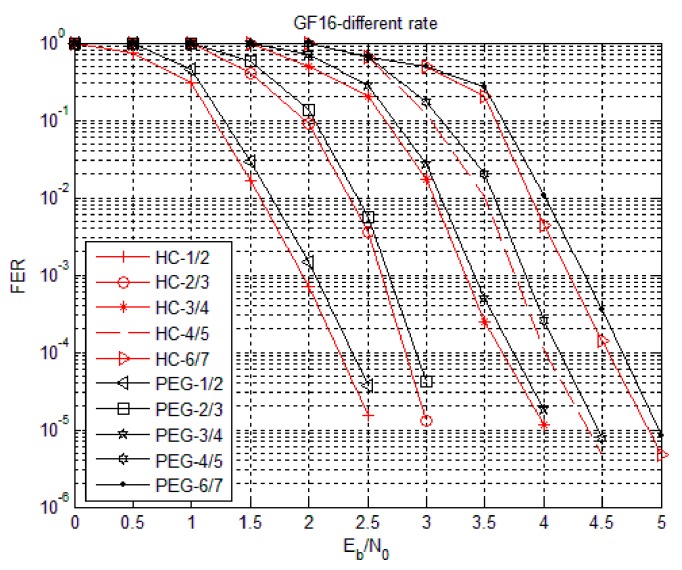
Frame Error Rate (FER) performance of HC algorithm with Code3.

The simulation parameters of the HC algorithm were set as follows. Using code rates 1/2, 2/3, 3/4, 4/5 and 6/7, the degree distribution was subject to the non-binary LDPC code of Equation (4) and the matrices are generated by PEG algorithm and HC algorithm. Simulation in hexadecimal (Code3) with fixed information bit length was also performed. In Code3, the base matrix under 1/2 code rate was 16 columns, the target matrix P was an unit array of 24 × 24, the base matrix under 2/3 code rate was 18 columns, the target matrix P was an unit array of 16 × 16, the base matrix under 3/4 code rate was 16 columns, the target matrix P was an unit array of 16 × 16, the base matrix under 4/5 code rate was 20 columns, the target matrix P was an unit array of 12 × 12, the base matrix under 6/7 code rate was 14 columns, the target matrix P was an unit array of 16 × 16 and fixed information bit length was 768 bits, which means the symbol length was 192. Figure 7, Figure 8 and Figure 9 show the Bit Error Rate (BER), Symbol Error Rate (SER) and Frame Error Rate (FER) performance of the PEG algorithm and HC algorithm under different code rates in the case of Code3, respectively.

From Figure 7, Figure 8 and Figure 9, the error rate of the non-binary LDPC code decreases with increasing $E_{b}/N_{0}$ and the performance of the non-binary LDPC code constructed by the HC algorithm was not significantly different from the non-binary LDPC code constructed by the PEG algorithm. The performance of the non-binary LDPC code constructed by the HC algorithm is better than the non-binary LDPC code constructed by the PEG algorithm under high SNR ratio. The HC algorithm combines the stochastic construction method with the structural construction method, which greatly reduces the complexity of hardware implementation and improves the error correction performance of the structured structure. Therefore, the check matrix construction algorithm of non-binary LDPC-CPM system in WSNs selects the proposed HC check matrix construction algorithm.

### 5.2. Information Bit Length of Non-Binary LDPC-CPM System in WSNs

**Figure 10:**
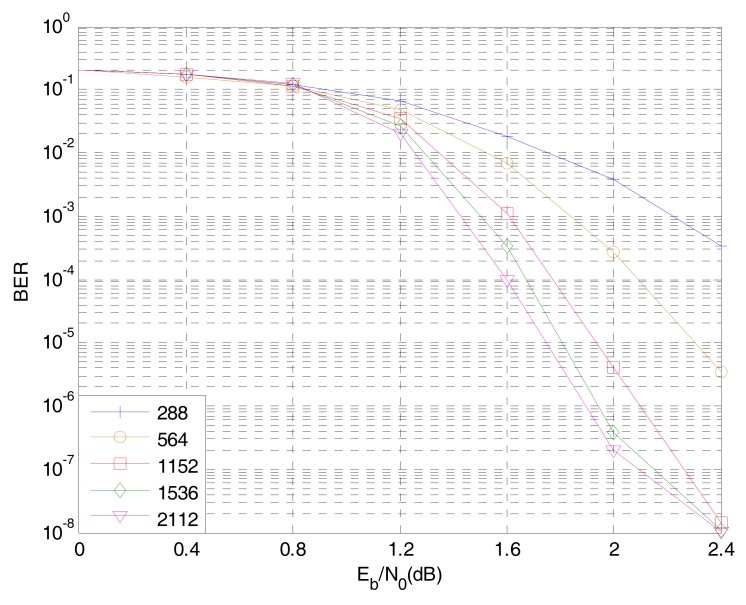
The influence of information bit length on the performance of 8-ary LDPC-CPM system.

Figure 10 shows the effect of the information bit length on the BER performance of an 8-ary LDPC-CPM system at 2/3 code rate. As can be seen from Figure 9, the BER performance of the system is gradually improved with the increase of the information bit length and the longer information bit length is, the better the system performance is. When the information bit length is 1536 bit, the base matrix is 6×18, the target matrix is 128×128, and the normalized signal-to-noise ratio $E_{b}/N_{0}$ is 2 dB, the BER reaches an order of magnitude of 10^−7^, which can meet the requirement of the reliability in WSNs and the complexity of the hardware implementation cannot be realized because the length of the code is too long.

### 5.3. The Code Rate of Non-Binary LDPC-CPM System in WSNs

**Figure 11:**
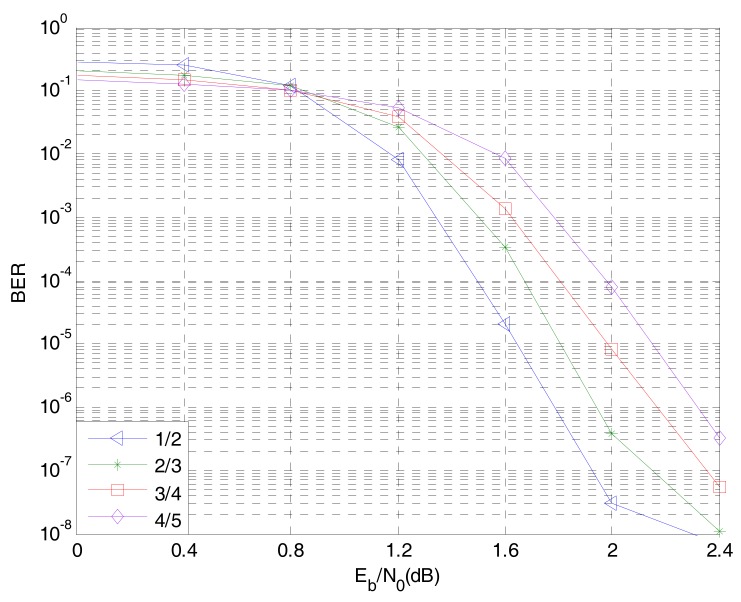
The influence of different code rate on the performance of 8-ary LDPC-CPM system.

Figure 11 shows the BER curves of an 8-ary LDPC-CPM system with code rates of 1/2, 2/3, 3/4 and 4/5. As can be seen from Figure 10, when the information bit length is 1536bit, the base matrix is 8×16, 6×18, 4×16, 4×20 respectively and the target matrix is 192×192, 128×128, 128×128, 96×96 respectively. The BER of the system is better, the BER performance of 1/2, 2/3 and 3/4 code rate can reach the expected target but the code rate is lower. The transmission rate of information also decreases, that is, the lower the band utilization rate of the system. Considering the reliability and transmission efficiency comprehensively, the code rate of the non-binary LDPC code is chosen to be 2/3.

### 5.4. Outer Iterations of Non-Binary LDPC-CPM System in WSNs

**Figure 12:**
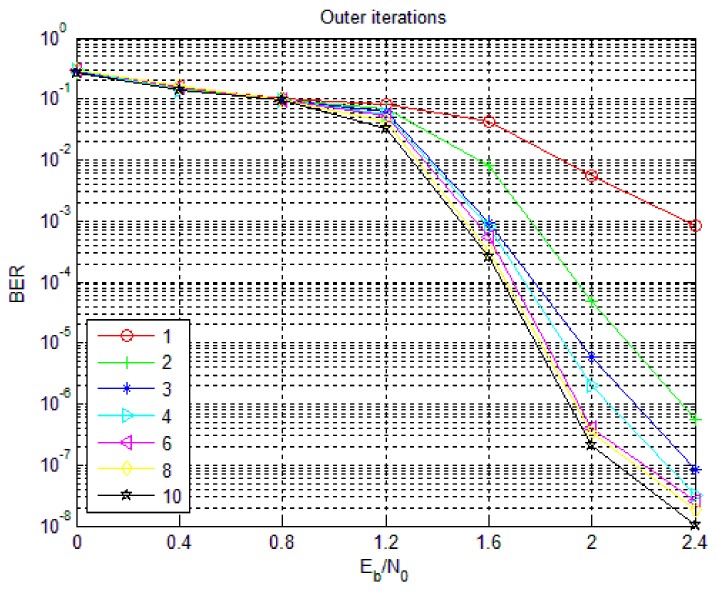
The influence of outer iterations on the performance of 8-ary LDPC-CPM system.

Non-binary LDPC-CPM system optimizes system performance through iterative demodulation and decoding, so the number of iterations is one of the key factors that determine system performance. The influence of the number of external iterations on system performance is examined below. In simulation, the bit length is 1536 bit and the code rate of LDPC is 2/3 and the number of iterations inside the LDPC decoder is fixed to 5 times. Figure 12 is the effect of the number of outer iterations on the BER performance of the 8-ary LDPC-CPM system. As can be seen from Figure 12, with the increase of the number of outer iterations, the BER of the system decreases and tends to converge and with the increase of the signal to noise ratio, the effect of the outer iteration on the BER is becoming more and more obvious. In the 0.5–1.2 dB region, the number of outer iterations has a very small effect on the BER but after 1.2 dB, the BER decreases rapidly with the increase of the number of iterations and converges after a certain number of iterations. After 6–8 iterations, the gain of the continuous iteration is very small. In order to reduce the delay of the system and reduce the complexity of hardware implementation, the number of outer iterations of the non-binary LDPC-CPM scheme is usually set to 8 times.

### 5.5. Inner Iterations of Non-Binary LDPC-CPM System in WSNs

**Figure 13:**
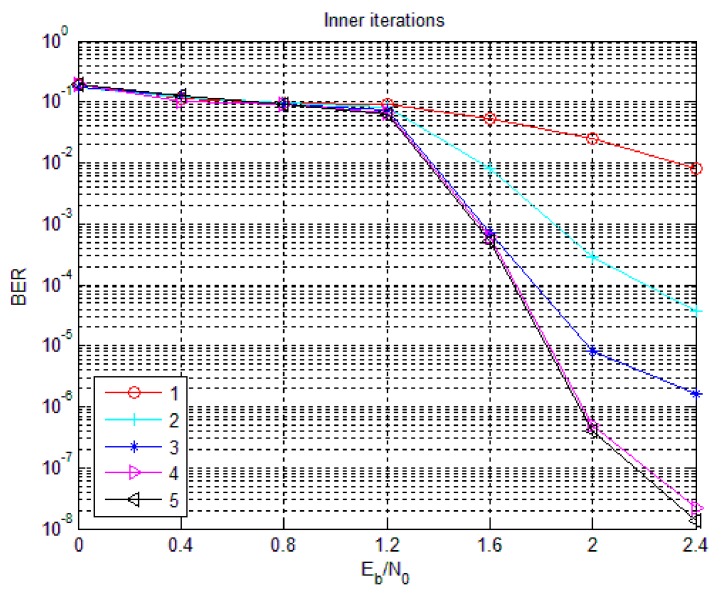
The influence of inner iterations on the performance of 8-ary LDPC-CPM system.

In the simulation, the information bit length is 1536 bits, the code rate of the non-binary LDPC code is 2/3 and the number of outer iterations is fixed at 8 times. Figure 13 shows the effect of the number of inner iterations on the BER performance of an 8-ary LDPC-CPM system. It can be seen from Figure 12, with the increase of the number of inner iterations, the BER of the system decreases and tends to converge and with the increase of the signal to noise ratio, the effect of inner iteration on the BER is becoming more and more obvious. In the 0–0.8 dB region, the number of inner iterations has a very small effect on the BER and the BER performance drops rapidly with the increase of the number of inner iterations and tends to converge after a certain number of iterations when the number of inner iterations increases. When the number of inner iterations is greater than or equal to 2, the BER performance of the system is much better than that of on internal iteration. However, after the number of inner iterations is greater than three, the system gain is very small. Since the number of iterations of non-binary LDPC decoding is the produce of the number of inner iterations and the number of outer iterations, in order to reduce the delay of the system and reduce the complexity of hardware implementation, the number of inner iterations of the non-binary LDPC-CPM scheme may be selected 4 times.

### 5.6. Energy Efficiency

**Figure 14:**
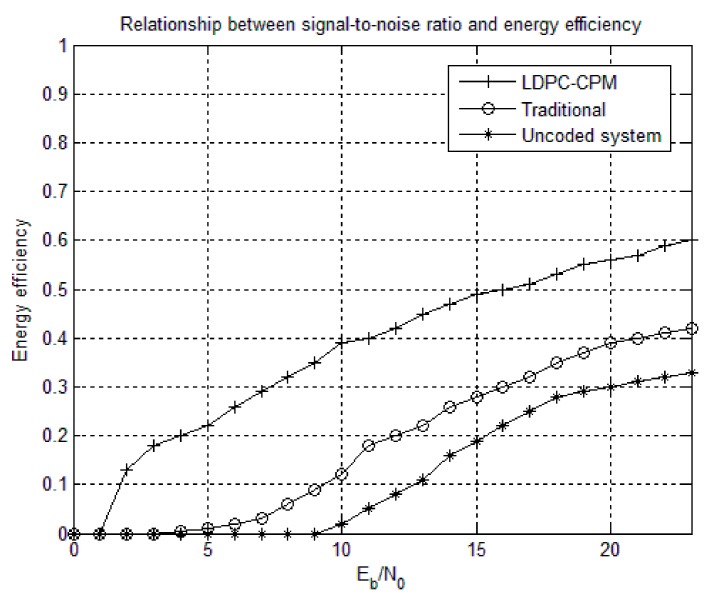
The relationship between signal-to-noise ratio (SNR) ratio and energy efficiency.

When communicating between two nodes, energy consumption occurs in two places, one is the transmission of information data (output power) and the other is the processing of data frames and error control. Therefore, the defined communication energy is equal to the sum of the energy consumed by the transmitted data and the energy consumed by the coding and decoding [34]. The design of the non-binary LDPC-CPM scheme parameters in the WSN is shown in Table 3. According to the simulation parameters in Table 3, the relationship between energy efficiency and Signal-to-noise (SNR) ratio is simulated. According to the above simulation diagram, when the BER of the system is required to be 10^–8^, the SNR ratio of non-binary LDPC-CPM system is 2.4 dB. The SNR ratio of the uncoded system is 8 dB. Under the same code rate and length, the traditional coding and decoding scheme can reach the index requirement at 6 dB [35,36,37]. Under the same conditions, the non-binary LDPC-CPM scheme can reduce the transmit power by 42–60% compared with traditional scheme and reduce the transmit power by 55–75% compared with the uncoded system. Thereby the relationship between energy efficiency and SNR ratio is obtained, as shown in Figure 14.

## 6. Encoding Complexity Analysis

Under the same error correction performance, the coding complexity was analyzed. According to the irPEG algorithm, the check matrix constructed by the algorithm had a lower triangular structure. Therefore, when the generated matrix was obtained, the computational complexity was lower, so the hardware was easier to implement. Based on improving the QC-LDPC algorithm, the irPEG algorithm was introduced to construct the base matrix. The obtained HC algorithm had no change in encoding complexity. However, the HC algorithm has a simpler matrix storage structure in terms of storage complexity than the irPEG algorithm. The encoding complexities of the PEG algorithm, irPEG algorithm and HC algorithm are shown in Table 4, where *w* is the average column weight of the generator matrix, *n* is the code length and *k* is the information bit length.

In terms of storage complexity, the HC algorithm is a structured construction method. The constructed non-binary LDPC code has a quasi-cyclic structure. When storing the matrix, only one p×p dimensional target matrix P, one J×L dimensional non-binary coefficient matrix $Gc_{J\times L}$ and one J×L cyclic shift coefficient matrix $S_{J\times L}$ are needed. However, a check matrix of the same size that was randomly constructed by the irPEG algorithm needs to store a check matrix of size p×J×p×L. The check matrix constructed by the HC algorithm has a simpler matrix storage structure than the irPEG algorithm. In addition, the HC algorithm has a certain code structure that overcomes the shortcomings of the long search time when the PEG algorithm and the irPEG algorithm construct the long code.

In terms of encoding complexity, Gaussian elimination coding algorithm was used for PEG algorithm and the non-binary superposition coding algorithm was used for the irPEG and HC algorithms. The encoding complexity calculation based on Gaussian elimination includes two parts: one to eliminate the check matrix into the structure of the lower triangular matrix, meaning the check matrix is preprocessed and the computational complexity is $o\left(n^{3}\right)$; and the other part is the encoding complexity $o\left(n^{2}\right)$. The complexity of the encoding depends on the sparsity of the generator matrix. The entire encoding process requires wn multiplication operations and (w−1)n addition operations. Even if the check matrix of the non-binary LDPC code is very sparse, the generator matrix is not sparse, the ratio of w and n cannot be ignored, so that the encoding complexity is proportional to the square of the code length. The complexity of the non-binary superposition coding algorithm depends entirely on the sparsity of the non-binary LDPC code check matrix. The entire encoding process requires (w−1)(n−k) addition operations and w(n−k) multiplication operations. When *w* can be seen as a small constant with respect to *n*, the coding algorithm has linear complexity.

The irPEG and HC algorithms proposed in this paper can directly construct a check matrix with a lower triangle that avoids the pretreatment of the check matrix and guarantees the sparseness of the matrix. Therefore, the average column weight *w* can be regarded as a small constant with respect to code length *n* and the linear complexity encoding of the non-binary LDPC code can be realized, which greatly reduces the encoding complexity compared with the conventional construction algorithm.

The improved algorithm decomposes the construction process and combines the advantages of the quasi-cyclic extension technique and the progressive edge construction method. It not only satisfies the need for degree distribution but also ensures the maximum requirement for the perimeter length. Its ring structure overlaps less, improving the speed of non-binary LDPC encoding. Additionally, the encoding is implemented with a set of shift registers and the encoding complexity is much lower than random construction. After the algorithm is extended to non-binary domains, the error correction capability is further improved.

## 7. Conclusions

We designed a data encoding scheme for WSNs and proposed a hybrid check matrix based on a non-binary LDPC code iterative coding algorithm in the design process. The algorithm uses backward superposition coding to decrease the triangular structure of the PEG algorithm and uses irPEG algorithm as the base matrix of the HC algorithm. Then, a cyclic shift matrix and a finite field coefficient matrix are generated by the improved QC-LDPC code algorithm with a lower triangular structure, which sets the number of the check matrix, from which the optimal check matrix is selected. The check matrix eliminates the influence of small rings, such as four-ring and forms a HC algorithm. Finally, specific parameters in a non-binary LDPC-CPM system for WSNs are given. The simulation results showed that the proposed algorithm has the randomness of a random construction algorithm, maintains the low coding complexity of the structured construction algorithm and reduces the loss caused by the structured construction algorithm to the error performance of a relatively compromised algorithm. Therefore, the non-binary LDPC-CPM scheme suitable for WSNs not only satisfies the requirements of the system for bit error rate but also effectively reduces the node transmit power, improves the energy usage rate and expands the node coverage. This will provide a new direction for a new direction for the development of WSNs in the future.

## Figures and Tables

**Table 1:** Comparison of the decoding time of the $E_{b}/N_{0}$ of Code1 at 3 dB.

Number of Erroneous Bits	Decoding Time (s)	
	PEG Algorithm	IrPEG Algorithm
10	1396	953
100	12,402	7153
1000	156,900	127,056

**Table 2:** Comparison of the decoding time of the $E_{b}/N_{0}$ of Code2 at 3 dB.

Number of Erroneous Bits	Decoding Time (s)	
	PEG Algorithm	IrPEG Algorithm
10	331	214
100	2452	1423
1000	20,510	9845

**Table 3:** The parameters of the non-binary LDPC-CPM system.

Check Matrix Construction Algorithm	HC Algorithm	Coding Algorithm	Non-Binary Iterative Algorithm
Decoding algorithm	Log-FFT-BP algorithm	Modulation	CPM
Inner iterations	4	Outer iterations	8
Degree distribution	$\lambda \left(x\right)=0.38354x+0.04237x^{2}+0.57409x^{3}$	Information bit	1536 bit
Code rate	2/3	q	8

**Table 4:** Encoding complexity analysis.

Matrix Construction Algorithm	Pretreatment	Additions Operations	Multiplication Operations	Encoding Complexity
PEG algorithm	$o\left(n^{3}\right)$	(w−1)n	wn	$o\left(n^{2}\right)$
IrPEG algorithm	**0**	(w−1)(n−k)	w(n−k)	o(n)
HC algorithm	**0**	(w−1)(n−k)	w(n−k)	o(n)
